# Effect of maternal age on maternal and perinatal outcomes including cesarean delivery following induction of labor in uncomplicated elderly primigravidae

**DOI:** 10.1097/MD.0000000000027063

**Published:** 2021-08-27

**Authors:** Yeonseong Jeong, Sung Pil Choo, Jisun Yun, Eui Hyeok Kim

**Affiliations:** Department of Obstetrics and Gynecology, National Health Insurance Service Ilsan Hospital, Goyang-si, South Korea.

**Keywords:** cesarean section, induced labor, maternal age, newborn infant, obstetric delivery

## Abstract

Age above 35 years at the time of birth is generally referred to as advanced maternal age (AMA), and it could be a risk factor for various complications besides genetic changes in the fetus. The primary outcome of this study was to determine if AMA is associated with emergent cesarean delivery (CD) following induction of labor (IOL). The secondary outcomes were a composite of adverse maternal and perinatal outcomes following IOL.

This retrospective observational study included women with singleton, live-born, cephalic, non-anomalous pregnancies undergoing IOL from 38 0/7 to 41 6/7 weeks of gestation. Mode of delivery and other maternal and neonatal outcomes were compared between women aged ≥35 (AMA) and <35 years. Multivariate logistic regression analyses were performed.

A total of 307 nulliparous women underwent IOL (≥35 years n = 73, 23.8%; <35 years n = 234, 76.2%) and among them, 252 (82.1%) delivered vaginally. The rate of CD was significantly higher in women of AMA (31.5% vs 13.7%, *P* = .001). Multivariable analysis showed that AMA was independently associated with CD (odds ratio 3.04, 95% confidence interval 1.55–5.96, *P* = .001). The rate of instrumental deliveries was higher in the AMA group (19.6% vs 8.2%, *P* = .043) and hemoglobin decrease during delivery was similar between the 2 groups (1.90 ± 1.25 vs 2.02 ± 1.27 mg/dL, all *P* > .05). Regarding neonatal outcomes, there was no difference between the 2 groups in the neonatal intensive care unit admission rate and Apgar score <7 at 5 minutes (30.3% vs 30.1% and 6.0% vs 8.2%, respectively, all *P* > .05). Neonatal intubation rate and severe respiratory problems were non-significantly higher in AMA (3.8% vs 2.7% and 3.4% vs 1.4%, respectively, all *P* > .05).

AMA was associated with an approximately three-fold increased likelihood of birth by CD and operative vaginal delivery in uncomplicated nulliparous women following IOL. However, we found no evidence that IOL in primigravid women of AMA increases adverse maternal and perinatal outcomes as compared with women aged <35 years except the high prevalence of CD and operative vaginal delivery.

## 1 Introduction

Age ≥35 years at the time of birth is referred to as advanced maternal age (AMA), which could be a risk factor for various kinds of complications besides fetal genetic changes. It was reported that hypertensive disease, gestational diabetes mellitus, placenta previa, placental abruption, and small-for-gestational age rates are higher among women aged ≥35 years than among younger women.^[1,2]^ It is especially well known that AMA is independently associated with an increased risk of stillbirth regardless of parity.^[3,4]^ Further, it has been reported that a woman's risk of having a stillbirth doubles at 35 years of age,^[5]^ and at 40 years of age, her risk is 3 times that of younger women.^[6]^

Induction of labor (IOL) is indicated in situations in which the outcomes for mothers and neonates are better if the pregnancy is not further prolonged.^[7,8]^ Therefore, considering IOL at term could be reasonable in women of AMA given the risk of stillbirth, besides indications such as hypertensive disease, diabetes, or intrauterine growth restriction. In fact, some studies suggested that induction on or before the due date in women aged ≥35 years may even be beneficial because the gestational age at delivery that is associated with the lowest cumulative risk of perinatal death is 38 weeks.^[9]^ Although induced labor remains controversial,^[10]^ the consensus is that labor induction increases the risk of cesarean delivery especially in nulliparous women.^[11–13]^

Childbearing at an AMA is becoming increasingly common in high-income countries.^[14,15]^ Currently in South Korea, about 30% women giving birth are aged ≥35 years, while in Australia, more than 1 in 5 women giving birth are aged ≥35 years.^[16]^ Furthermore, developments in artificial reproductive technologies may contribute to an increasing incidence of pregnancies in women outside the usual biological reproductive age range.

Women of AMA themselves typically think that their age puts their infant at an increased risk for a poor outcome.^[2]^ They tend to prefer a safer delivery mode, leading to an observed cesarean section rate of 38% and 50% among nulliparous women in the United Kingdom who are ≥35 and ≥40 years old, respectively.^[2]^ For both healthcare providers and women of AMA, scheduled induced labor even without a medical indication before 40 weeks is not an unreasonable option. Scheduled IOL has increasingly been adopted by women of AMA.^[17–19]^ Previously, we found that the risk of cesarean delivery was higher in women aged ≥35 years with a direct correlation between maternal age and failed induction,^[20]^ a finding that is consistent with those of previous studies.^[17,21]^ Risk-based counseling can strongly affect women's choices regarding management and intervention at term, so an understanding of the true risks in AMA may be crucial.

The aim of this study was to evaluate the effect of maternal age on cesarean delivery and maternal and perinatal outcomes following IOL in low-risk termed primigravid women to provide better information for pregnant women and healthcare providers who counsel them.

## 2 Materials and methods

### 2.1 Study population

**Figure 1:**
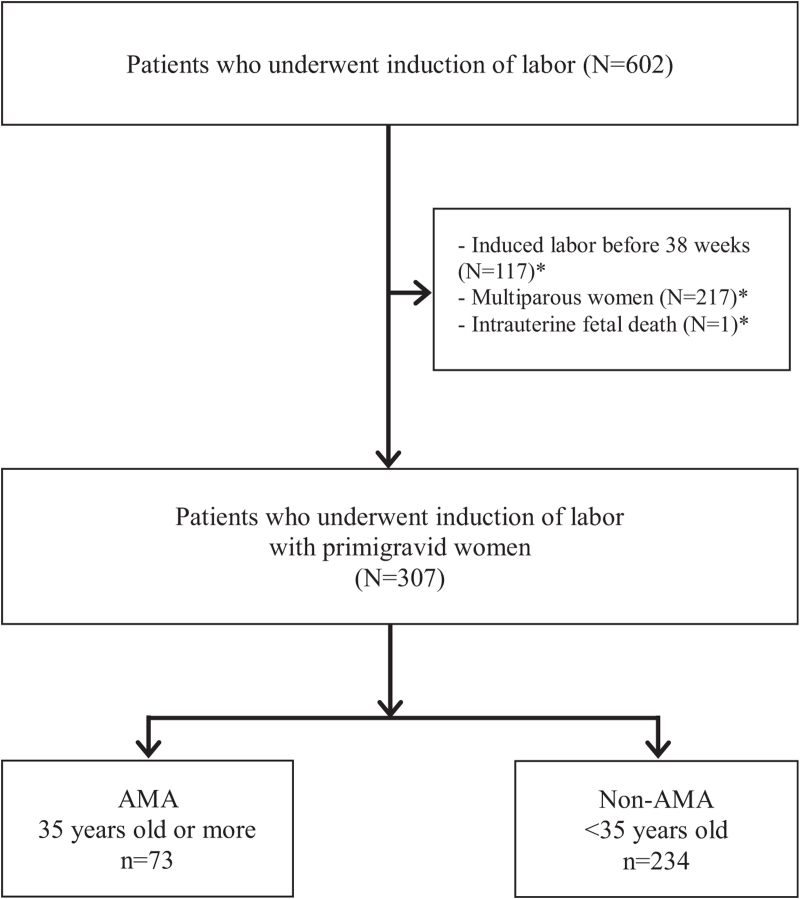
Flow chart of patient enrollment. ^∗^Per clinical assessment, there may be more than one indication per subject.

We performed a retrospective observational study of 602 women who underwent IOL at the National Health Insurance Service Ilsan Hospital in Goyang, Republic of Korea between January 2011 and December 2019. This study included only primigravidae with singleton pregnancies at term (from 38 0/7 to 41 6/7 weeks of gestation), and with vertex presentations. The exclusion criteria included fetal death, twin pregnancy, breech presentation, and severely anomalous infants. Among 602 women who were recruited in this study, 117 women who underwent induced labor before 38 weeks of gestation, 217 multiparous women, and 1 woman with intrauterine fetal death were excluded. The remaining 307 singleton pregnant women were included in this study (Fig. 1).

This study was approved by the institutional review board (#NHIMC 2019-03-010). The requirement for patient consent was waived since this was a retrospective study. Indications for labor induction were assessed according to the accepted criteria delineated in Obstetrics: Normal and Problem Pregnancies, 7th edition.^[22]^ These included postdate pregnancy (gestational age greater than 40+0 weeks), suspected intrauterine growth restriction, pregnancy-associated hypertension, oligohydramnios, uncontrolled diabetes mellitus or gestational diabetes mellitus, non-reassuring fetal status, other maternal medical conditions, fetal anomaly, and elective induction at maternal request or clinician's judgment.^[22]^ Elective labor induction was defined as IOL in patients without any maternal or fetal indications for delivery.^[11]^

Maternal and neonatal characteristics were obtained from the hospital‘s electronic medical records. Characteristics such as maternal age, maternal height, body mass index (BMI) just before delivery and before pregnancy, and gestational age were assessed. Neonatal characteristics including fetal birth weight and fetal engagement were assessed. Fetal engagement status was defined when the widest part of the baby's presenting part enters the pelvic brim or inlet. Women of AMA were defined as women aged ≥35 years at delivery. Cervical status was assessed using the Bishop score, transvaginal cervical length, and cervical funneling. Bishop's scores were calculated by assessing the following components: cervical dilatation, effacement, consistency, position, and station of the fetus; with a total score between 0 and 13.^[11]^ The maximum Bishop score for the study enrolment was 8. Cervical length and funneling were measured by transvaginal ultrasonography. Ultrasonography was performed by 1 expert using the Philips Ultrasound IU22 and EPIQ 7 (Bothell, WA, USA) with a vaginal probe. The transvaginal ultrasound examination was performed as described by Andersen et al.^[23]^ After an image of the cervix was obtained, the vaginal probe was withdrawn slightly until the image became blurry and was then reinserted carefully to avoid undue pressure on the cervix, which could increase the cervical length. The cervical longitudinal section was defined by the view of the cervical canal, and the cervical length was defined as the shortest value based on 4 or more measurements.^[23]^ Cervical funneling was defined by sonographic findings of the ballooning of the membranes into a dilated internal os, a closed external os, and at least 15% protrusion of the entire cervical length.^[24]^ To prevent bias, all assessments were performed after the decision to proceed with induction was made. To reduce inter-observer variation, these characteristics were measured by a single expert obstetrician (Eui Hyeok Kim) at a single hospital, with a single protocol.

IOL was performed using one of the 2 methods: intravenous oxytocin or intravaginal prostaglandin E2 insertion plus intravenous oxytocin. Patients with Bishop scores <4 were given intravaginal prostaglandin E2 before intravenous oxytocin was administered. Ten IU of intravenous was diluted in 1 L of 5% glucose solution and infused at an initial rate of 16 cc/h. The dosage was increased incrementally by 16 cc/h up to a maximum dose of 120 cc/h to achieve sufficient uterine contractions (more than 200 Montevideo units) according to the hospital's routine clinical protocol. For prostaglandin induction, an intravaginal prostaglandin E2 was inserted for a maximum of 12 hours until the Bishop's score became favorable (≥6) or until the membranes ruptured. Oxytocin was subsequently used after removal of the intravaginal prostaglandin E2 if sufficient uterine contractions were not present.

Cesarean delivery was performed if there was failure to progress, arrest of descent, fetal distress, prolonged second stage of labor, fever indicating chorioamnionitis, or a medical condition such as uncontrollable blood pressure, maternal request, or fetal face presentation. American College of Obstetricians and Gynecologists‘ guidelines were used to determine arrest of descent and prolonged second stage of labor.^[25]^

The primary maternal outcome measures and components were cesarean section rate, vacuum delivery rate, cesarean hysterectomy, uterine artery embolization due to postpartum bleeding, hemoglobin decrease during delivery, labor time from intervention, and the rate of labor time at ≥12 hour. Additionally, we evaluated maternal length of hospital stay, number of outpatient visits and readmission rate after discharge. Length of hospital stay was measured using the admission and discharge dates. The number of outpatient visits was defined as the number of times an individual visited the hospital within 50 days after the discharge date. This timeline was chosen because women usually have a routine visit at 6 weeks post-partum, and most postpartum complications occur within 50 days. We used readmission rates within 30 days post discharge because most instances of perioperative mortality and morbidity occur during this period, according to a previous study.^[26]^

The secondary perinatal outcome measures and components were perinatal death, meconium-stained amniotic fluid, Apgar score <7 at 5 minutes, fetal birth weight, respiratory distress syndrome, tachypnea of the newborn, hyaline membrane disease, neonatal intensive care unit admission rate, and intubation rate in the neonates.

### 2.2 Statistical analysis

Obstetric and neonatal data were analyzed and compared. Statistical analysis was performed using the Student *t* test for continuous variables and χ^2^ test and Fisher exact test for categorical variables to compare demographic and clinical characteristics between the vaginal and cesarean delivery groups. Independent predictors for successful vaginal delivery were determined by multivariate analysis using a logistic regression model. All *P* values were two-tailed and a *P* value <.05 was considered statistically significant. All analyzes were performed using the Statistical Package for Social Sciences, version 23.0 (IBM Corp., Armonk, NY).

## 3 Results

Among 602 women who were recruited in this study, 117 women who underwent induced labor before 38 weeks of gestation, 217 multiparous women, and 1 woman with intrauterine fetal death were excluded. The remaining 307 singleton pregnant primiparous women were included in this study (Fig. 1). Of the recruited 307 singleton primiparous women who underwent IOL at 38 weeks of gestation or more, 252 (82.1%) delivered vaginally, 73 (23.8%) were of AMA, and 234 (76.2%) were less than 35 years old. General maternal characteristics are shown in Table 1. Except for age, there were no differences in maternal characteristics including gestational weeks, BMI before pregnancy and at term, engagement status, and cervical status before induced labor between the 2 groups. There were no differences in indications of IOL except elective labor induction between the 2 groups as shown in Table 2.

**Table 1:** General maternal characteristics.

	AMA (n = 73)	Non-AMA (n = 234)	*P* value
Age (yrs old)	37.5 ± 7.3	29.5 ± 9.2	<.001^∗^
Gestational age (weeks)	39.1 ± 0.8	39.2 ± 0.8	.201
BMI before pregnancy (kg /m^2^)	22.4 ± 2.8	22.0 ± 2.0	.441
BMI at term (kg /m^2^)	27.4 ± 7.9	27.1 ± 7.2	.649
Weight gain during pregnancy	12.8 ± 2.3	13.3 ± 3.3	.492
Engagement	57 (78.1)	195 (83.3)	.300
Bishop score^†^	3.5 ± 1.8	3.7 ± 1.7	.227
Cervix length (mm)	19.5 ± 8.2	18.6 ± 8.1	.838

Values are presented as mean ± standard deviation, or the number (%). AMA = advanced maternal age, BMI = body mass index.

∗ Statistical significance.

† Total possible score =13.

**Table 2:** Indications of induction of labor.

	AMA (n = 73)	Non-AMA (n = 234)	*P* value
Oligohydramnios	9 (12.3)	21 (9.0)	.376
Post-due date	4 (5.5)	11 (4.7)	.760
High BP/ preeclampsia	8 (11.0)	16 (6.8)	.316
DM / GDM	4 (5.5)	8 (3.4)	.489
PROM	9 (12.3)	19 (8.1)	.350
Elective	26 (35.6)	125 (53.4)	.011^∗^
IUGR	8 (11.0)	26 (11.1)	1.00
Non-reassuaring NST	0	2 (0.9)	1.00
Maternal condition^†^	4 (5.5)	6 (2.6)	.256
Minor fetal anomaly^‡^	1 (1.4)	0	.238

Values are presented as the number (%) AMA, advanced maternal age; BP, blood pressure; DM = diabetes mellitus, GDM = gestational diabetes mellitus, PROM = premature rupture of membranes, IUGR = intrauterine growth restriction, NST = non stress test.

∗ Statistical significance.

† Maternal request for induced labor/ liver enzyme elevation.

‡ Fetal hydronephrosis.

Table 3 shows the primary maternal outcomes. The rate of cesarean delivery was significantly higher in women of AMA than in non-AMA women (31.5% vs 13.7%; *P* = .001). Moreover, operative vaginal delivery with a vacuum was significantly higher in the AMA group (16.9% vs 8.2%; *P* = .043). Incidence of uterine artery embolization and the rate of postpartum transfusion due to massive postpartum bleeding were similar between the 2 groups. Hemoglobin decrease after delivery was also similar. The length of postpartum hospital stay was higher in the AMA group (4.2 ± 1.3 days vs 4.6 ± 1.6 days; *P* = .014.). Other characteristics including postpartum bleeding and delivery time were similar between the 2 groups (Table 3). The number of women with more than 2 outpatient visits within 50 days after discharge and readmission rate within 30 days after discharge were similar between the 2 groups (24.7% vs 22.2% and 1.4% vs 3.8%, respectively, all *P* > .05) (Table 3).

**Table 3:** Primary maternal outcomes.

	AMA (n = 73)	Non-AMA (n = 234)	*P* value
Cesarean section rate	23 (31.5)	32 (13.7)	.001^∗^
Vacuum delivery	12 (16.9)	19 (8.2)	.043^∗^
Uterine A. Embolization	1 (1.4)	1 (0.4)	.413
Postpartum Transfusion	3 (4.1)	7 (3.0)	.783
Hgb decrease during delivery (g/dl)	2.02 ± 1.27	1.90 ± .9 5	.459
Time for Intervention to deliver (min)^†^	928 ± 639	883 ± 538	.607
Delivery time ≥12 h^†^	20 (40.8)	96 (47.5)	.428
Length of stay (d)	4.6 ± .26	4.2 ± 1.3	.014^∗^
Outpatient visits >2 within 50 d after discharge	18 (24.7)	52 (22.2)	.632
Readmission within 30 d after discharge	1 (1.4)	9 (3.8)	.461

Values are presented as the number (%) or mean ± standard deviation. AMA = advanced maternal age, Hgb = Hemoglobin, Uterine A. = uterine artery.

∗ Statistical significance.

† Included only vaginal delivery.

The perinatal outcomes and components are shown in Table 4. Fetal weight and the rate of fetal weight <2500 g were similar between the 2 groups. Although the rate of 1-minutes Apgar score <7 was higher in the AMA group (52.1% vs 34.2%, *P* = .006), other outcomes, including 5-minutes Apgar score <7, meconium-stained amniotic fluid, neonatal intensive care unit admission rate, and intubation rate were similar between the 2 groups (Table 4). Severe respiratory problems including respiratory distress syndrome, hyaline membrane disease, and tachypnea of the newborn were very rare and the differences in occurrences between the 2 groups were not significant.

**Table 4:** Secondary perinatal outcome and components.

	AMA (n = 73)	Non-AMA (n = 234)	*P* value
Fetal birth weight (g)	3186 ± 447	3198 ± 461	.848
Fetal weight <2500 g	5 (6.8%)	12 (5.1%)	.564
Meconium AF	12 (16.4)	35 (15.0)	.852
AS <7 at 1 min	38 (52.1)	80 (34.2)	.006^∗^
AS <7 at 5 min	6 (8.2)	14 (6.0)	.586
RDS and TTN	1(1.4)	8 (3.4)	.691
NICU admission rate	22 (30.1)	71 (30.3)	1.000
Intubation rate	2 (2.7)	9 (3.8)	1.000

Values are presented as mean ± standard deviation or the number (%). AF = amniotic fluid, AMA = advanced maternal age, AS = Apgar score, NICU = neonatal intensive care unit, RDS = respiratory distress syndrome, TTN = transient tachypnea of newborn.

∗ Statistical significance.

Reasons for emergent cesarean delivery during induced labor are showed in Table 5. Reasons for cesarean delivery were fetal distress, chorioamnionitis, failure to progress, prolonged second stage, unstable lie (the frequent changing of fetal lie and presentation), and maternal requests, and the differences between the 2 groups were not significant (Table 5).

**Table 5:** Reasons for cesarean delivery.

	AMA (n = 23)	Non-AMA (n = 32)	*P* value
Fetal distress	7 (30.4)	5 (15.6)	.208
Chorioamnionitis	0 (0)	3 (9.4)	.257
Failure to progress	12 (52.2)	18 (56.3)	.790
Prolonged 2nd stage	3 (13.0)	4 (12.5)	1.000
Unstable lie	0 (0)	1 (3.1)	1.000
Maternal request during labor induction	1 (4.3)	1 (3.1)	1.000

Values are presented as the number (%) AMA = advanced maternal age.

Multivariate logistic regression analysis adjusted for maternal BMI, gestational age, Bishop score, premature rupture of membranes and engagement, showed that AMA was independently associated with cesarean delivery (odds ratio [OR], 3.04; 95% confidence interval [CI], 1.55–5.96; *P* = .001) and operative vaginal delivery with vacuum (OR, 2.72; 95% CI, 1.21–6.12; *P* = .015), as shown in Table 6.

**Table 6:** Logistic regression analysis.

	Cesarean delivery			Vacuum delivery		
	OR	95% CI	*P* value	OR	95% CI	*P* value
AMA	3.04	1.55–5.96	.001^∗^	2.72	1.21–6.12	.015^∗^

Adjusted for maternal body mass index, gestational age, Bishop score, premature rupture of membranes and fetal engagement. CI = confidence interval, OR = odds ratio.

∗ Statistical significance.

## 4 Discussion and conclusions

This study showed that AMA was an independent predictor for cesarean delivery and operative vaginal delivery with vacuum following IOL at term in uncomplicated nulliparous women. However, IOL did not increase other adverse short-term effects on maternal and perinatal outcomes in women of AMA compared to non-AMA women (those with younger maternal age), in this study. Regardless of maternal age, the absolute rate of poor pregnancy outcome may be low and most primigravidae of AMA delivered without severe adverse maternal and neonatal complications following IOL.

Other studies also reported an association between AMA and higher risk of adverse maternal and infant outcomes.^[27–29]^ The reason for the similar perinatal outcomes between the 2 groups is that most adverse fetal outcomes are attributed to fetal aneuploidy, which are associated with missed abortion in early pregnancy. However, our study included termed pregnancy only and did not include pregnancy loss in early pregnancy. Regarding maternal outcomes, cesarean deliveries may be a risk factor for the slightly higher incidence of maternal morbidities, rather than vaginal deliveries. Further, though the rate of cesarean delivery was higher in women of AMA in our study, there were no significant differences between groups in severe maternal outcomes including cesarean hysterectomy, the rate of transfusion, and uterine embolization due to massive postpartum transfusion. Besides, time for intervention of IOL to delivery and the rate of delivery time ≥12 hour were not different between the 2 groups, and outpatient visits **>**2 within 50 days after discharge and readmission within 30 days after discharge were also not different.

Multivariate logistic regression analysis adjusted for maternal BMI, gestational age, Bishop score, premature rupture of membranes and engagement showed that AMA was independently associated with cesarean delivery and operative vaginal delivery with vacuum in this study, which is consistent with previous studies.^[21,30,31]^ There are several hypotheses to explain the association between AMA and dystocia. Collagen progressively replaces normal muscle in the walls of myometrial arteries in nongravid mothers of AMA, and almost every segment of every artery is affected to some degree.^[32]^ Restriction of the luminal expansion of arteries by these lesions obstructing and decreasing blood flow to the placenta in AMA may be 1 factor that contributes to this fetal jeopardy and nonprogressive labor.^[32]^ Women of AMA, therefore, may be less likely to tolerate the hemodynamic demands of both pregnancy and labor. Furthermore, a gradual decrease in myometrial function with advancing maternal age may lead to less effective uterine contractions.^[33,34]^ Finally, the presence of medical comorbidities may compound the reduced physiological tolerance that women of AMA have to labor; thus, a relative increase in cesarean delivery or operative vaginal delivery may be unavoidable.^[34]^

This study has several strengths. First, we fully analyzed the maternal and neonatal conditions through electronic medical records in a single institution. Second, we applied the same protocol of induced labor in the same institution. Finally, we included only nulliparous women with low-risk pregnancies at term, because the risk of stillbirth is especially higher among nulliparous women than among multiparous women in all maternal age groups.^[35,36]^ Parity and being in the high-risk group could be 2 of the most important risk factors for cesarean delivery following IOL.^[37,38]^

The limitations of this study included a small population size and the heterogeneous nature of the indications for labor induction. Moreover, obstetricians’ and women's perceptions about AMA as being dangerous may have influenced the earlier decision for cesarean delivery without proceeding with labor despite the small risk during IOL.

According to another previous study, IOL can decrease the risk of stillbirth in AMA^[39]^ and can also reduce the chance of sudden disruption of a patient's life and provider's work. However, if elective IOL is considered in AMA pregnancies at term, inherent risks must be discussed, and informed consent associated with cesarean delivery and operative vaginal delivery must be obtained.

In conclusion, AMA was associated with an approximately three-fold increased likelihood of birth by cesarean delivery and operative vaginal delivery in uncomplicated nulliparous women following IOL. However, we found no evidence that induced labor in primigravid women of AMA increases adverse maternal and perinatal outcomes as compared with non-AMA women except the high prevalence of cesarean delivery and operative vaginal delivery. We believe our findings could help providers give appropriate guidance to women of AMA, with respect to the choice of mode of delivery and possible maternal and neonatal outcomes.

## Author contributions

**Conceptualization:** Eui Hyeok Kim.

**Data curation:** Eui Hyeok Kim.

**Methodology:** Eui Hyeok Kim.

**Supervision:** Eui Hyeok Kim, Sung Pil Choo, Jisun Yun.

**Validation:** Eui Hyeok Kim, Sung Pil Choo.

**Writing – original draft:** Yeonseong Jeong, Eui Hyeok Kim.

**Writing – review & editing:** Yeonseong Jeong, Eui Hyeok Kim.
